# Impact of delivery mode on diastasis recti abdominis, pelvic floor muscle function, and quality of life in early postpartum women: a cross-sectional study

**DOI:** 10.3389/fmed.2026.1845766

**Published:** 2026-07-06

**Authors:** Yilin Liu, Wenqin Huang, Zhiliang Zhang, Luyao Wang, Xinyi Wu, Lu Chen, Yan Xia

**Affiliations:** Department of Postpartum Plastic and Reconstructive Surgery, Shanghai First Maternity and Infant Hospital, Shanghai, China

**Keywords:** caesarean section, diastasis recti abdominis, pelvic floor, postpartum period, quality of life, sleep

## Abstract

**Background:**

The early postpartum period is critical for maternal recovery. This study aimed to investigate the impact of delivery mode on diastasis recti abdominis (DRA), pelvic floor muscle strength, sleep quality, and overall quality of life at 6–8 weeks postpartum.

**Methods:**

A cross-sectional study was conducted among 196 primiparous postpartum women, including 100 vaginal deliveries and 96 caesarean sections. DRA severity was assessed by measuring the inter-recti distance using clinical palpation and high-frequency ultrasound. Pelvic floor muscle strength was evaluated using a perineometer. Sleep quality and quality of life were assessed using the Pittsburgh Sleep Quality Index and the WHOQOL-BREF questionnaire, respectively.

**Results:**

Women who underwent caesarean section exhibited a higher prevalence of DRA (*p* = 0.019) and wider inter-recti distance at the umbilicus and supra-umbilical levels compared to the vaginal delivery group with differences reaching statistical significance (all *p* < 0.010). The vaginal delivery group demonstrated significantly weaker fast-twitch pelvic floor muscle fibres (*p* = 0.036). Furthermore, the caesarean section group reported significantly lower scores in the physical (*p* = 0.017) and psychological (*p* = 0.003) domains of quality of life. Multiple linear regression identified poor sleep quality and caesarean section, rather than the severity of DRA, as strong independent factors of reduced quality of life.

**Conclusion:**

Delivery mode is associated with distinct postpartum recovery trajectories. Caesarean section is associated with more severe DRA and poorer early postpartum quality of life, whereas vaginal delivery is specifically associated with weaker fast-twitch pelvic floor muscles. Sleep quality is a primary determinant of maternal wellbeing, highlighting the need for delivery-mode-tailored, multidisciplinary postpartum rehabilitation with sleep support as an integral component of care.

## Introduction

The postpartum period represents a critical phase of profound anatomical, physiological and psychological transition. Among the numerous musculoskeletal changes experienced during this period, diastasis recti abdominis (DRA), defined as the abnormal widening of the linea alba, is one of the most prevalent yet frequently overlooked conditions. DRA leads to abdominal wall laxity, compromised core stability, and potential secondary impairments in pelvic floor function and postural alignment (1). Based on established ultrasonographic criteria, DRA is typically diagnosed when the inter-rectus distance (IRD) exceeds 14 mm at 3 cm above the umbilicus, 20 mm at the umbilicus, or 2 mm at 3 cm below the umbilicus (2). Epidemiological evidence indicates that approximately 33.1% of women are affected at 21 weeks of gestation, with the prevalence peaking at 60.0% by 6 weeks postpartum and subsequently declining to 45.4 and 32.6% at 6 and 12 months postpartum, respectively (3). This trajectory highlights the period from 6 weeks to 6 months postpartum as a critical window for characterizing DRA and identifying its modifiable and non-modifiable determinants.

Delivery mode is a key determinant that may differentially influence postpartum abdominal wall recovery. Caesarean section (CS) involves direct surgical disruption of abdominal wall layers, potentially altering fascial integrity and muscle architecture during the healing process. In contrast, vaginal delivery (VD) causes significant fluctuations in intra-abdominal pressure, which directly affects the tension of the rectus abdominis muscles and the linea alba (4, 5). Research on the postpartum consequences of different delivery modes has predominantly concentrated on pelvic floor function, including vaginal contractility, pelvic floor muscle strength, and sexual health outcomes. Existing evidence generally suggests that CS exerts a lesser impact on early postpartum pelvic floor function relative to VD, although these differences tend to attenuate over time (6, 7), and that the type and severity of perineal trauma sustained during VD further influence the risk of postpartum dyspareunia and the timing of sexual activity resumption (8, 9) By comparison, studies specifically addressing the relationship between delivery mode and DRA remain sparse and inconclusive. Although prior investigations have compared pelvic floor muscle strength between women with and without DRA (10) or reported that CS primarily alters abdominal fascia and muscle thickness whereas VD predominantly affects the muscular layer (4), a systematic comparison of the prevalence, severity, and clinical manifestations of DRA stratified by delivery mode has yet to be conducted.

The clinical significance of DRA extends well beyond aesthetic concerns. Emerging evidence suggests that DRA is associated with pelvic organ prolapse, reduced abdominal muscle strength, lower back pain, and impaired health-related quality of life (QoL) (11, 12). Moreover, early postpartum QoL is inherently multifactorial; conditions such as sleep disturbances, which are highly prevalent among new mothers, may compound the physical burden of DRA and further compromise maternal wellbeing. Although several risk factors for persistent DRA have been identified, including elevated body mass index (BMI), multiparity, multiple gestations, and gestational diabetes (13), the existing literature has predominantly focused on the later postpartum stages, leaving a notable gap in our understanding of the factors influencing DRA during the crucial early recovery period.

Therefore, this cross-sectional study aimed to comprehensively compare the severity of DRA, pelvic floor muscle strength, sleep quality, and QoL between women who underwent VD and those who delivered by CS at six to 8 weeks postpartum. We hypothesized that delivery mode would significantly and differentially influence the postpartum recovery trajectory, with CS primarily exacerbating abdominal wall dysfunction and VD predominantly affecting pelvic floor integrity, and that these distinct physical sequelae would differentially impact early postpartum QoL.

## Methods

### Study design and participants

This comparative cross-sectional study was conducted at the Department of Postpartum Plastic and Reconstructive Surgery, Shanghai First Maternity and Infant Hospital between October 2025 and January 2026. The study protocol was approved by the Institutional Review Board (Approval No. KS25439), and written informed consent was obtained from all participants prior to enrolment.

The study population consisted of postpartum women attending their routine postnatal check-ups. Participants were grouped into two groups based on their delivery mode as the vaginal delivery (VD) and the caesarean section (CS).

### Eligibility criteria

The inclusion criteria were primiparous women aged 20–45 years with a singleton pregnancy, approximately 6–8 weeks postpartum, and no history of prior abortion. The exclusion criteria included multiple pregnancies, history of previous abdominal surgeries other than caesarean section, severe systemic diseases, cognitive impairment, inability to complete the questionnaires, and congenital abdominal wall defects.

### Sample size calculation

The sample size was calculated using G*Power software (version 3.1.9.7, Germany). According to a previous study (14), the incidence of DRA was reported to be 77.7% in women with vaginal delivery and 92.1% in women with caesarean section. Using a two-sided significance level (𝛼) of 0.05 and a statistical power (1 − 𝛽) of 80%, the minimum required sample size was calculated to be 93 participants per group. To account for a potential 5% rate of missing data or invalid questionnaire responses, the total planned sample size was approximately 196 participants.

### Clinical and demographic data collection

Baseline demographic and obstetric characteristics were collected from electronic medical records and patient interviews. These included age, body mass index (BMI), gestational days at delivery, days postpartum at the time of assessment, neonatal birth weight, education level, history of prenatal urinary leakage, and history of postpartum exercise.

The primary outcome of this study was DRA severity, as quantified by inter-recti distance (IRD). The secondary outcomes included pelvic floor muscle function, health-related QoL, and sleep quality.

DRA was evaluated using both finger assessment (palpation) and ultrasound imaging. All measurements were performed by a single experienced physiotherapist to eliminate inter-rater variability. For the finger assessment, participants were placed in a supine position with knees bent at 90 degrees and feet flat on the examination bed. They were instructed to perform a partial sit-up, raising their head and shoulders off the bed. The examiner used their fingers to palpate and measure the width of the gap between the medial borders of the rectus abdominis muscles. High-frequency ultrasound (Model E20 pro; Guangzhou Huibo Information Technology Co., Ltd., Guangzhou, China) was used as the objective standard. The IRD was measured in millimetres (mm) at four specific anatomical levels: 5 cm above the umbilicus, 3 cm above the umbilicus, at the umbilicus, and 3 cm below the umbilicus.

Pelvic floor muscle function was assessed using a standard perineometer or biofeedback device. Participants were instructed to perform specific pelvic floor muscle contractions. The assessment recorded the maximum burst power (measured in mmHg), Type I muscle fibre strength (sustained endurance contractions), and Type II muscle fibre strength (rapid, short contractions).

The World Health Organization Quality of Life Brief Version (WHOQOL-BREF) was used to assess QoL. It comprises 26 items categorized into four domains: physical health, psychological health, social relationships, and environmental health, with higher scores indicating a better QoL. The Pittsburgh Sleep Quality Index (PSQI) was utilized to evaluate sleep quality over the past month. It yields a global score ranging from 0 to 21, with higher scores indicating poorer sleep quality.

Statistical analyses were performed using SPSS (version 26.0, IBM Corp., Armonk, NY, USA). Continuous variables were assessed for normality using the Shapiro–Wilk test and were presented as mean ± standard deviation (SD) or median (interquartile range). Comparisons were made using the independent samples *t*-test or the Mann–Whitney *U* test, as appropriate. Categorical variables were presented as frequencies and percentages and compared using the Chi-square test. To identify factors independently associated with IRD and QoL, multiple linear regression models were constructed. Variables considered clinically relevant were included as covariates in the adjusted models. Unstandardized coefficients (*B*) and confidence intervals (CIs) were calculated. A two-sided *p*-value < 0.05 was considered statistically significant.

## Results

A total of 196 women were included in this study and divided into the VD group (*n* = 100) and the CS group (*n* = 96). The demographic and clinical characteristics were presented in Table 1. There were no differences between two groups in terms of age, BMI, days postpartum, neonatal birth weight, education level, and history of prenatal urinary leakage (*p* > 0.05). However, the median gestational days were slightly shorter in the CS group compared to the VD group (*p* = 0.040).

**Table 1:** General characteristics.

Characteristics	Vaginal delivery group (*n* = 100)	Caesarean section group (*n* = 96)	*p*-value
Age, years, mean ± SD	30.55 ± 3.36	31.23 ± 3.45	0.164
BMI, kg/m^2^, mean ± SD	22.82 ± 2.83	23.70 ± 3.90	0.074
Gestational days, median (P25, P75)	276.0 (270.5, 280.0)	274.0 (270.0, 277.75)	**0.040** ^*^
Days Postpartum, median (P25, P75)	47.0 (43.0, 62.5)	47.5 (43.25, 69.75)	0.322
Neonatal birth weight, g, mean ± SD	3,189.00 ± 412.43	3,283.85 ± 373.74	0.094
Education level, *n* (%)			0.219
College or below	15 (15.0)	12 (12.5)	
Bachelor’s degree	46 (46.0)	55 (57.3)	
Postgraduate degree	39 (39.0)	29 (30.2)	
History of prenatal urinary leakage, *n* (%)	19 (19.0)	15 (15.6)	0.533

^*^indicates *p* < 0.05.

Applying the > 20 mm threshold, the overall prevalence of DRA was 76.0% (149/196). Notably, the prevalence was significantly higher in the CS group compared to the VD group (83.3% vs. 69.0%; *p* = 0.019). Anatomical assessment of the abdominal wall revealed distinct differences between the groups (Table 2). Ultrasound evaluation showed that the CS group had significantly wider IRD at the umbilicus (*p* < 0.001) and at supra-umbilical levels (3 cm: *p* = 0.005; 5 cm: *p* < 0.001). The median IRD at the umbilicus was 27.00 mm in the CS group versus 22.00 mm in the VD group. These findings were consistent with finger assessments, which also indicated significantly wider gaps in the CS group at the umbilicus and supra-umbilical levels (*p* < 0.01). The VD group showed significantly weaker Type II muscle fibre strength compared to the CS group (*p* = 0.036). No significant differences were observed in Type I muscle fibre strength or maximum burst power. In terms of QoL, the CS group had significantly lower scores in the physical domain (*p* = 0.017) and the psychological domain (*p* = 0.003). There were no significant differences in the social relationships and environmental domains.

**Table 2:** Comparison of pelvic floor muscle strength, diastasis recti, and quality of life between groups.

Variables	Vaginal delivery group (*n* = 100)	Caesarean section group (*n* = 96)	Statistic (*t*/*Z*)	*p*-value
Pelvic floor muscle strength				
Max burst power, mmHg, median (P25, P75)	63.00 (54.00, 76.50)	66.50 (57.00, 81.75)	−1.400	0.162
I fibre	1.00 (0.00, 5.00)	3.00 (0.00, 5.00)	−1.176	0.239
II fibre	5.00 (3.00, 5.00)	5.00 (3.25, 5.00)	−2.099	**0.036** ^*^
DRA—Finger assessment, cm, median (P25, P75)				
5 cm above umbilicus	12.00 (6.00, 16.13)	15.00 (9.38, 18.00)	−2.741	**0.006** ^*^
3 cm above umbilicus	15.00 (10.88, 18.00)	18.00 (13.88, 22.50)	−3.021	**0.003** ^*^
At umbilicus	7.00 (6.00, 8.50)	8.50 (6.50, 10.00)	−3.510	**<0.001** ^*^
3 cm below umbilicus	8.25 (4.88, 15.00)	12.00 (6.00, 15.00)	−0.267	0.789
DRA—Ultrasound assessment, cm, median (P25, P75)				
5 cm above umbilicus	12.90 (9.13, 17.10)	16.25 (12.95, 21.25)	−3.974	**<0.001** ^*^
3 cm above umbilicus	16.55 (11.85, 20.48)	19.50 (14.33, 23.73)	−2.786	**0.005** ^*^
At umbilicus	22.00 (18.60, 27.43)	27.00 (21.63, 37.55)	−3.750	**<0.001** ^*^
3 cm below umbilicus	11.75 (7.38, 16.38)	13.60 (9.53, 17.03)	−1.705	0.088
PSQI, median (P25, P75)	11.00 (9.00, 13.00)	10.00 (8.00, 12.00)	−1.343	0.179
WHOQOL-BREF, mean ± SD				
Physical	12.57 ± 1.57	12.07 ± 1.35	2.418	**0.017** ^*^
Psychological	12.97 ± 1.73	12.28 ± 1.47	3.005	**0.003** ^*^
Social relationships domain	15.59 ± 2.04	15.08 ± 2.09	1.708	0.089
Environmental domain	14.83 ± 1.65	14.55 ± 1.38	1.264	0.208

^*^Indicates *p* < 0.05; DRA, diastasis recti abdominis; PSQI, Pittsburgh Sleep Quality Index.

Multiple linear regression analysis was performed to identify independent factors of IRD shown in Table 3 and Supplementary Table S1. Adjusting for potential confounding factors such as age, BMI, days postpartum, neonatal birth weight, exercise history, gestational days, and breastfeeding status, delivery mode remained a significant factor of IRD. Caesarean section was associated with an increased IRD of 3.71 mm at 5 cm above the umbilicus (*p* < 0.001), 3.46 mm at 3 cm above the umbilicus (*p* = 0.020), 5.35 mm at the umbilicus (*p* < 0.001), and 1.95 mm at the 3 cm below the umbilicus (*p* = 0.038). Additionally, postpartum days were positively associated with IRD at the umbilicus (*p* = 0.045), and a history of postpartum exercise was associated with increased IRD at 3 cm above the umbilicus (*p* = 0.010).

**Table 3:** Multiple linear regression analysis of the association between delivery mode and IRD at different anatomical levels.

Measurement sites	Unadjusted model		Adjusted model	
	*B* (95% CI)	*p*-value	*B* (95% CI)	*p*-value
Supra-umbilical 5 cm	3.60 (1.90, 5.30)	**<0.001**	3.71 (1.96, 5.45)	**<0.001** ^*^
Supra-umbilical 3 cm	3.81 (0.99, 6.62)	**0.008**	3.46 (0.55, 6.37)	**0.020** ^*^
At umbilicus	5.28 (2.56, 8.00)	**<0.001**	5.35 (2.50, 8.20)	**<0.001** ^*^
Infra-umbilical	1.53 (−0.23, 3.28)	0.087	1.95 (0.11, 3.79)	**0.038** ^*^

^*^Indicates *p* < 0.05.

Adjusted for Age, BMI, postpartum days, neonatal birth weight, History of postpartum exercise, gestational days, and breastfeeding status.

To further confirm whether DRA severity contributed to the lower QoL observed in the CS group, multiple linear regression models were constructed for the physical and psychological domains (Table 4; Supplementary Table S2). After adjusting for delivery mode, sleep quality and other covariates, IRD was not significantly associated with physical QoL (*p* = 0.739) or psychological QoL (*p* = 0.868). Instead, the regression analysis identified sleep quality and delivery mode as the primary determinants of QoL. Poor sleep quality was strongly associated with lower scores in both the physical domain (*p* < 0.001) and psychological domain (*p* = 0.005). Caesarean section was independently associated with lower scores in both physical (*p* = 0.012) and psychological domains (*p* = 0.002). Additionally, older age was a negative factor for the physical domain (*p* = 0.020).

**Table 4:** Multiple linear regression analysis of the association between IRD and quality of life.

Outcomes	Unadjusted model		Adjusted model	
	*B* (95% CI)	*p*-value	*B* (95% CI)	*p*-value
WHOQOL—Physical	−0.01 (−0.03, 0.01)	0.307	0.004 (−0.017, 0.024)	0.739
WHOQOL—Psychological	−0.018 (−0.041, 0.005)	0.131	−0.002 (−0.026, 0.022)	0.868

^*^Indicates *p* < 0.05.

Adjusted for Delivery mode, Age, BMI, Postpartum days, sleep quality (PSQI), History of urinary incontinence, and History of postpartum exercise.

## Discussion

This cross-sectional study comprehensively examined the impact of delivery mode on DRA severity, pelvic floor muscle function, quality of life, and sleep quality at 6–8 weeks postpartum. The principal findings demonstrate a clear divergence in early postpartum recovery profiles according to the delivery mode. Women who underwent caesarean section exhibited significantly higher prevalence of DRA and wider inter-recti distance at the umbilicus and supra-umbilical levels, as confirmed by both clinical palpation and high-frequency ultrasound, and reported poorer physical and psychological QoL compared to those who had a vaginal delivery. In contrast, vaginal delivery was associated with significantly weaker Type II (fast twitch) pelvic floor muscle fibres. Multiple linear regression analyses further identified caesarean section and poor sleep quality, rather than the anatomical severity of DRA itself, as the primary independent factors of reduced QoL in this early postpartum cohort.

Both ultrasound and clinical palpation assessments consistently demonstrated that the CS group had significantly wider IRD at the umbilicus and supra-umbilical levels compared to the VD group. After adjusting for age, BMI, postpartum days, neonatal birth weight, and exercise history, CS remained an independent factor of increased IRD, with estimated increases of 3.71 mm, 3.46 mm, 5.35 mm, and 1.95 mm at 5 cm above, 3 cm above, at the umbilicus, and 3 cm below, respectively. These findings extend the current evidence by quantifying the magnitude of IRD differences between delivery modes across multiple anatomical levels using standardized ultrasound measurements.

The mechanistic basis for this association likely relates to the direct surgical disruption of the anterior abdominal wall during caesarean delivery. The fascial incision, combined with lateral retraction of the rectus abdominis muscles to access the uterus, disrupts the structural integrity of the myofascial unit and initiates an inflammatory healing cascade that may impede the natural retraction of the linea alba during the early postpartum period (4). Moreover, postoperative pain and protective guarding behaviors may inhibit voluntary engagement of the deep core musculature, further delaying the restoration of abdominal wall tension. In contrast, although vaginal delivery generates substantial fluctuations in intra-abdominal pressure that transiently stress the linea alba (5), it preserves the anatomical continuity of the anterior abdominal wall, potentially facilitating earlier recovery of fascial tension. Although the absolute differences in IRD between the groups (approximately 3–5 mm) may appear modest, they are clinically meaningful in the context of early postpartum recovery. This magnitude of delayed fascial restoration in the CS group highlights a critical window where targeted conservative management could prevent the long-term persistence of DRA and its associated musculoskeletal impairments.

Notably, the between-group differences were confined to the umbilical and supra-umbilical regions, with no significant difference observed at the infra-umbilical level. This spatial pattern is anatomically plausible. Above the arcuate line, the linea alba is comparatively wider and constitutes the sole anterior fascial covering of the rectus sheath, rendering it more susceptible to both mechanical distension during pregnancy and surgical perturbation during caesarean delivery. The infra-umbilical region of the linea alba, by contrast, contains a greater proportion of transversely oriented collagen fibres and benefits from the additional structural reinforcement provided by the posterior rectus sheath, which together confer a greater capacity to resist tensile stresses (15, 16).

The pelvic floor muscles comprise both slow-twitch (Type I) and fast-twitch (Type II) fibres; Type I fibres provide tonic support, whereas Type II fibres generate the rapid contractions essential for maintaining urethral closure during sudden rises in intra-abdominal pressure (10). During vaginal delivery, the fetal head subjects the levator ani to extreme stretching, causing direct myofibre disruption or avulsion and pudendal neuropraxia, significantly increasing the long-term risk of stress urinary incontinence (17–19). Type II fibres are particularly susceptible to such stretch-induced injury, and vaginal parity has been shown to alter pelvic floor muscle fibre architecture through chronic remodelling (20). The preferential loss of Type II fibre function compromises the rapid contractile response needed to counteract abrupt pressure increases, predisposing women to stress urinary incontinence even when tonic Type I support remains intact. Clinically, this specific impairment is highly relevant, as it translates directly to a compromised ability to maintain continence during sudden physical exertion, such as coughing, sneezing, or lifting the newborn. These findings align with prospective evidence that vaginal delivery is associated with significantly higher rates of stress urinary incontinence and other pelvic floor disorders compared with caesarean section (17). However, the selective nature of the deficit described above, limited to Type II fibres rather than global pelvic floor weakness, suggests that early postpartum screening and fibre-type-specific pelvic floor training following vaginal delivery merit further investigation.

In the multiple linear regression models, delivery mode and neonatal birth weight emerged as independent factors of wider supra-umbilical IRD. The association with neonatal birth weight is mechanistically intuitive, as a larger fetus generates greater mechanical distension of the anterior abdominal wall during the third trimester, resulting in more pronounced stretching of the linea alba that may persist postpartum. These findings are broadly consistent with a narrative review (21), which identified parity, BMI, and diabetes as the most plausible risk factors for DRA, and highlighted that the true prevalence and determinants of DRA remain inadequately characterized due to heterogeneous measurement methods and diagnostic criteria across studies. Postpartum days showed a significant negative association with IRD at the umbilicus, reflecting gradual spontaneous resolution consistent with the temporal trajectory (3). An unexpected finding was the positive association between a history of postpartum exercise and wider supra-umbilical IRD (*B* = 4.489, *p* = 0.010). Given the cross-sectional design, this may reflect reverse causality, whereby women who perceived a more prominent abdominal gap were more motivated to initiate early exercise. Certain early postpartum exercises, particularly traditional abdominal crunches that generate elevated intra-abdominal pressure, may inadvertently exacerbate DRA when performed without professional supervision (22). Prospective studies with detailed characterization of exercise modality and timing are needed to clarify the directionality of this relationship. The overall explanatory power of the regression models was modest (*R*^2^ = 0.011–0.093), suggesting that additional unmeasured factors, such as connective tissue composition, genetic predisposition, and collagen metabolism, likely play important roles in determining DRA severity and warrant investigation in future studies.

A clinically important finding is that the CS group reported significantly lower scores in both the physical and psychological domains of the WHOQOL-BREF. Regression analysis confirmed that caesarean section and poor sleep quality were strong, independent factors of reduced QoL after adjusting for IRD, age, BMI, and other covariates. The diminished physical QoL in the CS group likely reflects postoperative pain, restricted mobility, and newborn care demands superimposed upon surgical recovery. The lower psychological scores may relate to unmet birth expectations and perceived loss of control during delivery (23). While the absolute differences in WHOQOL-BREF scores between the groups are relatively small, they reflect clinically important disparities in daily functioning and early maternal wellbeing, further justifying the need for early pain management and psychological support following surgical delivery. Older age was an additional negative factor for the physical domain, possibly reflecting diminished physiological resilience with advancing maternal age. Poor sleep quality emerged as a particularly potent factor of reduced QoL in both the physical and psychological domains. Notably, the two groups did not differ significantly in PSQI scores, indicating that the quality-of-life disparity is not attributable to differential sleep disruption. However, sleep quality appears to exert a strong, group-independent influence on maternal wellbeing, consistent with evidence that postpartum sleep disturbance is a central, modifiable determinant of maternal recovery (24).

The severity of DRA was not significantly associated with QoL in either the unadjusted or adjusted models, suggesting that at 6–8 weeks postpartum, women’s perceived QoL is primarily shaped by systemic factors such as pain, sleep deprivation, and overall physical recovery rather than by the anatomical magnitude of abdominal separation. These findings support the development of delivery-mode-tailored postpartum rehabilitation pathways. Caesarean delivery warrants early pain management, sleep hygiene counseling, and guided abdominal wall rehabilitation emphasizing deep core activation while avoiding high-load exercises that may exacerbate DRA (1). Following vaginal delivery, early pelvic floor screening with attention to Type II fibre strength is recommended. Regardless of delivery mode, integration of sleep support and psychological screening into routine postnatal care is strongly advised.

The strengths of this study include the comprehensive, multi-dimensional assessment of postpartum recovery, combining validated subjective instruments with objective evaluations using both clinical palpation and high-frequency ultrasound for DRA assessment and perineometer for pelvic floor muscle function. The use of a single experienced examiner for all physical assessments minimized inter-rater variability, and the adequate sample size ensured sufficient statistical power for the primary between-group comparisons.

However, several limitations must be acknowledged. First, the cross-sectional design precludes the establishment of causal relationships. Therefore, the findings represent associations rather than direct causations, and any broader clinical implications derived from them should be interpreted with caution. This is particularly relevant for the observed association between postpartum exercise and wider IRD. The assessment was confined to the early postpartum period, and longitudinal follow-up is required to determine whether the observed between-group differences persist over time. Second, due to the lack of pre-pregnancy baseline data, it is not possible to rule out the possibility that pre-existing differences between groups, such as pre-pregnancy DRA status, pelvic floor function, body weight and exercise history. Third, several potential confounding variables were not collected, including gestational weight gain, prenatal exercise, postpartum pain, and psychological status, which may have introduced residual confounding. Additionally, the distinction between elective and emergency caesarean section was not recorded, which precludes a more nuanced assessment of how delivery indication may influence postpartum recovery outcomes. Fourth, the pelvic floor assessment in this study was limited to measurements of muscle strength. The absence of a more comprehensive assessment may have limited the completeness of our pelvic floor outcome data and the breadth of our clinical recommendations. Finally, the generalizability of the findings to other populations and healthcare settings may be limited, and multi-centre prospective cohort studies are warranted to validate and extend these results.

## Conclusion

In conclusion, the delivery mode significantly shapes the trajectory of early postpartum physical recovery. Caesarean section is associated with more severe diastasis recti abdominis at the umbilical and supra-umbilical levels and with poorer physical and psychological quality of life, whereas vaginal delivery is specifically associated with weaker the fast-twitch fibres of the pelvic floor muscles. Sleep quality, rather than the anatomical severity of the abdominal separation, emerges as a primary determinant of maternal wellbeing at 6–8 weeks postpartum. These findings underscore the critical need for personalized, multidisciplinary postpartum rehabilitation programs tailored to the distinct anatomical and psychological challenges associated with each delivery mode, with sleep support as an integral component of care for all postpartum women.

## Data Availability

The original contributions presented in the study are included in the article/Supplementary material, further inquiries can be directed to the corresponding author.
