# Supplementary material for: Impact of delivery mode on diastasis recti abdominis, pelvic floor muscle function, and quality of life in early postpartum women: a cross-sectional study
Source: Front Med (Lausanne). 2026 Jul 6;13:1845766. doi: 10.3389/fmed.2026.1845766 (PMC13381517; doi:10.3389/fmed.2026.1845766)
Supplement: Supplementary file 1 [file Data_Sheet_1.PDF]

Table S1 Multiple linear regression analysis of factors associated with inter-rectus distance at different anatomical levels.

	**Supra-umbilical (5cm)**		**Supra-umbilical (3cm)**		**At Umbilicus**		**Infra-umbilical**	
	B (95% CI)	P-value	*B* (95% CI)	P-value	*B* (95% CI)	P-value	*B* (95% CI)	P-value
**Delivery Mode (Cesarean)**	3.63 (1.92, 5.34)	**<0.001**	3.84 (1.01, 6.68)	**0.008**	5.23 (2.47, 7.99)	**<0.001**	1.61 (-0.17, 3.40)	0.077
**Age**	0.02 (-0.24, 0.28)	0.882	0.08 (-0.35, 0.50)	0.718	0.08 (-0.33, 0.50)	0.689	0.02 (-0.25, 0.29)	0.898
**BMI**	-0.24 (-0.49, 0.02)	0.073	-0.27 (-0.70, 0.16)	0.212	-0.09 (-0.51, 0.32)	0.657	-0.20 (-0.47, 0.07)	0.148
**Postpartum Days**	-0.03 (-0.06, 0.001)	0.060	-0.04 (-0.09, 0.01)	0.105	-0.05 (-0.10, -0.01)	**0.026**	-0.02 (-0.05, 0.01)	0.140
**Neonatal Birth Weight**	0.003 (0.000, 0.005)	**0.019**	0.003 (-0.001, 0.01)	0.093	0.003 (-0.001, 0.01)	0.162	0.001 (-0.001, 0.004)	0.201
**History of Postpartum Exercise**	1.21 (-0.83, 3.25)	0.245	4.37 (0.99, 7.76)	**0.012**	2.26 (-1.04, 5.55)	0.178	0.73 (-1.41, 2.86)	0.502
R^2	0.130		0.086		0.105		0.039	
Note: * indicates P < 0.05								

Table S2 Multiple linear regression analysis of factors associated with quality of life at different anatomical levels.

	**WHOQOL - Physical**		**WHOQOL - Psychological**	
	B (95% CI)	P-value	*B* (95% CI)	P-value
**Delivery Mode**	-0.544 (-0.965, -0.123)	**0.012**	-0.754 (-1.238, -0.270)	**0.002**
**Age**	-0.071 (-0.131, -0.011)	**0.020**	-0.016 (-0.085, 0.053)	0.652
**BMI**	-0.028 (-0.087, 0.031)	0.354	-0.020 (-0.087, 0.048)	0.565

**Postpartum Days**	0.002 (-0.005, 0.009)	0.564	0.004 (-0.004, 0.012)	0.348
IRD	0.004 (-0.017, 0.024)	0.739	-0.002 (-0.026, 0.022)	0.868
**PSQI**	-0.168 (-0.239, -0.098)	**<0.001**	-0.116 (-0.198, -0.035)	**0.005**
**History of urinary incontinence**	0.200 (-0.415, 0.815)	0.522	-0.209 (-0.916, 0.498)	0.561
**History of Postpartum Exercise**	-0.229 (-0.705, 0.248)	0.345	-0.216 (-0.764, 0.332)	0.437
R^2	0.169		0.097	
Note: * indicates $P < 0.05$				
